# The Role of Nasal Microbiota and Sensitivity in Patients With Chronic Rhinosinusitis at a Rural Tertiary Care Hospital

**DOI:** 10.7759/cureus.76048

**Published:** 2024-12-19

**Authors:** Bosco Suriya Luke Rathnakumar, Ujval Gowda, Charuvi Guttal, Subrumuthuram Gautham

**Affiliations:** 1 Department of Otolaryngology, Head and Neck Surgery, Sri Devaraj Urs Academy of Higher Education and Research, Kolar, IND

**Keywords:** aminoglycosides, antibiotics, cephalosporins, culture, diagnostic nasal endoscopy, lund-kennedy score, macrolides, pseudomonas, sensitivity, snot-22

## Abstract

Background and aim

Etiopathogeneses of chronic rhinosinusitis are poorly understood. Recent research emphasizes culture-independent molecular sequencing to identify clusters of flora that may function as drivers of inflammation. Studies also indicate that macrolides are as effective as corticosteroids in controlling chronic rhinosinusitis. In this study, we aimed to isolate microbial cultures from the middle meatus of patients suffering from chronic rhinosinusitis and assess the isolates for similarities and antibiotic sensitivity. We also sought to identify the pathogenic species disrupting the nasal microbiome and provide appropriate antibiotics based on the least minimum inhibitory concentration (MIC) from the cultures. Disease progression and response to treatment were evaluated using the Sino-Nasal Outcome Test-22 (SNOT-22) and the Lund-Kennedy endoscopy scoring system. Specifically, diagnostic nasal endoscopy (DNE) was performed in patients with chronic rhinosinusitis without nasal polyps (CRSnNP), and the pre-treatment Lund-Kennedy score was recorded, along with subjective data collected from the SNOT-22 questionnaire. After isolating cultures from the middle meatus, antibiotic treatment was provided based on these findings. A repeat DNE was conducted to calculate the post-treatment Lund-Kennedy score and collect the post-treatment SNOT-22 score. Finally, the pre- and post-treatment scores were compared to assess any statistically significant differences.

Methods

The patients upon arrival to the hospital and diagnosed with chronic rhinosinusitis without nasal polyposis (CRSnNP) based on the joint EPOS 2020 Criterion for the same were enrolled in the study. The patients were administered an SNOT-22 Questionnaire for subjective evaluation. The patients underwent a diagnostic nasal endoscopy (DNE) to calculate the Lund-Kennedy score, take swabs from the middle meatus for culture and sensitivity, and provide objective evaluation by the assessing physicians. The scores were recorded at the first visit and on each visit till the two weeks of antibiotic treatment were completed. The patients were treated with antibiotics as per the cultured isolates. The Lund-Kennedy scores and SNOT-22 scores before and after treatment were compared to note the response to treatment.

Results

The mean average Lund-Kennedy score and SNOT-22 scores dropped following a course of antibiotics. The patients also experienced symptomatic relief. The most commonly isolated organism among the samples evaluated was *Pseudomonas aeruginosa*. The best response to antibiotics was noted with aminoglycosides. Total resistance (100%) to macrolides and amoxicillin was also observed, which contradicts the antibiotic guidelines of EPOS 2020, ICAR 2021, and JTFPP 2014.

Conclusion

This study found that the invasive species disrupting the local nasal microbiome of the participants consisted of various pathogenic microorganisms. It indicated that a culture-based treatment of CRSnNP will yield better results compared to empirical antibiotics. The present study also suggests revising guidelines for antibiotic use and developing personalized antibiograms for treating chronic rhinosinusitis.

## Introduction

The European Position Paper on Rhinosinusitis and Nasal Polyposis, 2020 defines Chronic Rhino Sinusitis (CRS) in adults as presence of two or more symptoms, one of which should be either nasal blockage/obstruction/congestion or nasal discharge (anterior/posterior nasal drip) with/without facial pain/pressure, reduction or loss of smell with evidence of mucosal inflammation which is to be correlated further by endoscopic or radiological findings of duration greater than 12 weeks [1]. The incidence of CRS in the United States is estimated to be 2.1% [2], 2.1-4.3% in Europe [3], and about 134 million Indians suffer from chronic sinusitis [4]. It is estimated to affect up to 12% of the world’s population and thus poses a huge burden on the healthcare system [3].

Nasal cavities were believed to be sterile environments and the presence of any organism was considered pathological which has now been disproven [3]. Both the mucosal and epithelial surfaces of the sino-nasal tract are colonized by aerobic and anaerobic flora. The core microbiome is defined as organisms that are shared across unrelated individuals. According to the International Sino-nasal Microbiome Study, which is the largest study on the microbiota of the sino-nasal tract, the sino-nasal mucosa plays host to several aerobic commensals like *Staphylococcus aureus*, α-hemolytic streptococci, *Streptococcus pneumoniae*, *Haemophilus influenzae*, coagulase-negative Staphylococci[3]. A study carried out on a series of patients afflicted with acute rhinosinusitis revealed that as the patient progresses from an acute to chronic illness, the microbiome turns anaerobic from aerobic and facultative species [5]. Recent studies on nasal microbiota have also shown that there are differences between the microbiota of healthy nasal mucosa and those with chronic rhinosinusitis [6]. The human microbiome project is probably the largest study undertaken to quantify nasal microbiome from the swabs of anterior nares. The microbiome of the nasal cavity confirmed the presence of *Staphylococcus aureus*, *Staphylococcus epidermidis*, and Corynebacteriaceae genera [7]. Langille et al. reported a predominance of Corynebacterium in the nasal microbiome from the middle meatus in nearly 50% of the study sample. Corynebacterium and Propionibacterium, followed by Staphylococcaceae, were isolated in decreasing order of abundance [8].

Studies conducted on the nasal microbiome of patients with chronic rhinosinusitis with or without polyposis and control groups showed the prevalence of Citrobacteronly among patients without nasal polyposis [9]. This has led to the development of the "Microbiome hypotheses" of chronic rhinosinusitis which states that the nasal microbiome contributes to the pathogenesis of chronic rhinosinusitis by Th1 or Th2-mediated inflammatory response. It further adds that the nasal microbiome produces superantigens that promote a strong T cell-mediated localized immune response leading to the production of Th2-mediated cytokines (IL-4, IL-5, IL-13). This causes the proliferation of eosinophils and B-cell activation resulting in the superantigen-specific IgE antibody production creating a positive feedback cycle of Th2-mediated inflammation. Therefore, it can be sufficiently expostulated that antibiotic regimens may have a role to play in the treatment of chronic rhinosinusitis [10-12]. Antibiotics, especially macrolides, have been shown to provide treatment outcomes similar to inhalational corticosteroids.

## Materials and methods

Study design, period, and data source

This was a prospective observational study conducted over a period of three months. Patients with chronic rhinosinusitis, aged between 18 and 65 years, who met the inclusion criteria at the Department of Otorhinolaryngology, Head and Neck Surgery, RL Jalappa Hospital and Research Center, attached to Sri Devaraj Urs Medical College, Kolar, Karnataka, were included in the study. Based on a study conducted by Gwak et al. and the prevalence of CRS in India at 0.02%, with an alpha error of 80%, the sample size was calculated to be 80 [7].

Inclusion and exclusion criteria

Eighty patients, aged between 18 and 65 years, treated at RL Jalappa Hospital, Tamaka, Kolar, Karnataka, with clinically proven CRSnNP were included in the study. Exclusion criteria included patients with nasal vestibulitis, a recent history of nasal surgery, allergic rhinitis, or fungal sinusitis.

Methodology

Owing to the nature of the disease, the patients presented at the outpatient department of our hospital. After clinical diagnosis based on the EPOS 2020 criteria, the patients were enrolled in the study. Once clinical diagnosis of chronic rhinosinusitis was established, the patients were evaluated for the severity of the disease by the SNOT-22 Questionnaire. The patients underwent routine evaluation including a thorough examination of the ear by otoendoscopy; nose and nasopharynx by a diagnostic nasal endoscopy. Under endoscopic guidance, the middle meatus was identified. The endoscopy was performed by two physicians who were in no way shape or form affiliated with the study and were blinded to the participants. They scored the patients by the Lund-Kennedy score. Mucoid/mucopurulent secretions from the middle meatus were collected on sterile nasal swabs and cultured on nutrient agar within 30 minutes of collection of swabs. Antibiotic sensitivity of cultures was checked using the Kirby-Bauer disk diffusion method. The patients were administered antibiotics with the least minimum inhibitory concentration (MIC) based on the cultures isolated. Patients were followed up over a course of two weeks and the response to therapy was observed by a repeat diagnostic nasal endoscopy and assessing the nasal cavity by the Lund-Kennedy score (Table 1) and objective evaluation of the patients’ symptoms by the SNOT-22 Questionnaire (Table 2) [13,14]. This was done to eliminate any recall bias during the SNOT-22 Questionnaire. Both pre- and post-treatment evaluations were done by the same attending physicians. The obtained data were entered and assessed by the SPSS version 29 (Armonk, NY: IBM Corp.). The SNOT-22 scores and Lund-Kennedy scores before and after antibiotics were compared by the Wilcoxon signed-rank test.

**Table 1:** Lund-Kennedy endoscopic grading system.

Characteristics	Score definition
Nasal polyps	0 = none; 1 = confined to middle meatus; 2 = beyond middle meatus
Discharge	0 = none; 1 = clear and thin; 2 = thick and purulent
Edema	0 = absent; 1 = mild; 2 = severe
Scarring	0 = absent; 1 = mild; 2 = severe
Crusting	0 = absent; 1 = mild; 2 = severe

**Table 2:** Sino-Nasal Outcome Test-22 (SNOT-22). The most important items affecting the patients' health are to be marked on the right of each question (maximum of five items). The date and patients' unique hospital identification number are to be mentioned at the top of the questionnaire sheet. SNOT-22 was developed from the modification of SNOT-20 by the National Comparative Audit of Surgery for Nasal Polyposis and Rhinosinusitis Royal College of Surgeons of England.

Considering how severe the problem is when you experience it and how often it happens, please rate each item below on how "bad" it is by circling the number that corresponds with how you feel using this scale.	No problem	Very mild problem	Mild or slight problem	Moderate problem	Severe problem	Problem as bad as it can be
1. Need to blow nose	0	1	2	3	4	5
2. Nasal blockage	0	1	2	3	4	5
3. Sneezing	0	1	2	3	4	5
4. Runny nose	0	1	2	3	4	5
5. Cough	0	1	2	3	4	5
6. Post-nasal discharge	0	1	2	3	4	5
7. Thick nasal discharge	0	1	2	3	4	5
8. Ear fullness	0	1	2	3	4	5
9. Dizziness	0	1	2	3	4	5
10. Ear pain	0	1	2	3	4	5
11. Facial pain/pressure	0	1	2	3	4	5
12. Decreased sense of smell/taste	0	1	2	3	4	5
13. Difficulty falling asleep	0	1	2	3	4	5
14. Wake up at night	0	1	2	3	4	5
15. Lack of a good night’s sleep	0	1	2	3	4	5
16. Wake up tired	0	1	2	3	4	5
17. Fatigue	0	1	2	3	4	5
18. Reduced productivity	0	1	2	3	4	5
19. Reduced concentration	0	1	2	3	4	5
20. Frustrated/restless/irritable	0	1	2	3	4	5
21. Sad	0	1	2	3	4	5
22. Embarrassed	0	1	2	3	4	5

## Results

Observations and results

Eighty patients were clinically diagnosed with chronic rhinosinusitis, of which, 44 were males (55%) and 36 were females (45%). The distribution of patients in each specific age group is provided in Table 3. In either sex, patients between 25 and 45 years of age appeared most afflicted.The number of males exceeded the females in terms of incidence of the disease (Table 3)butin each age group, the females seem to be the worst affected in terms of severity of the disease based on* *Lund-Kennedy and SNOT-22 scores(Tables 4, 5)*.*

**Table 3:** Distribution of patients based on age groups.

Age group (years)	Males	Females
18-25	11	9
25-45	22	18
45-65	11	6
>65	0	3

**Table 4:** Antibiotics given as per sensitivity. Our study focussed on oral antibiotics. Hence, we chose azithromycin among macrolides, amoxicillin with clavulanic acid among penicillins, gentamicin among aminoglycosides, ciprofloxacin/levofloxacin among fluoroquinolones as per the sensitivity. As none of the patients were sensitive to macrolides or penicillins, they have not been reflected in the table.

Age group (years)	Antibiotic	Males	Females
18-25	Aminoglycosides	3	4
	Fluoroquinolones	8	5
25-45	Aminoglycosides	6	9
	Fluoroquinolones	16	9
45-65	Aminoglycosides	6	2
	Fluoroquinolones	5	4
>65	Aminoglycosides	0	2
	Fluoroquinolones	0	1

**Table 5:** Average Lund-Kennedy scores pre- and post-antibiotic treatment. SD: standard deviation; w: Wilcoxon rank

Age group (years)	Average Lund-Kennedy scores (mean±SD)					
	Males			Females		
	Pre-treatment score	Post-treatment score	w, p-value	Pre-treatment score	Post-treatment score	w, p-value
18-25	3±1	2±1	3, 0.008	3±1	2±1	0, 0.04
25-45	3±1	1±1	46, 0.001	4±1	2±1	34, 0.003
45-65	3±1	1±1	0, 0.0033	4±1	1±1	0, 0.001
>65	0	0	0	4±1	1±1	0, 0.001

**Figure 1:**
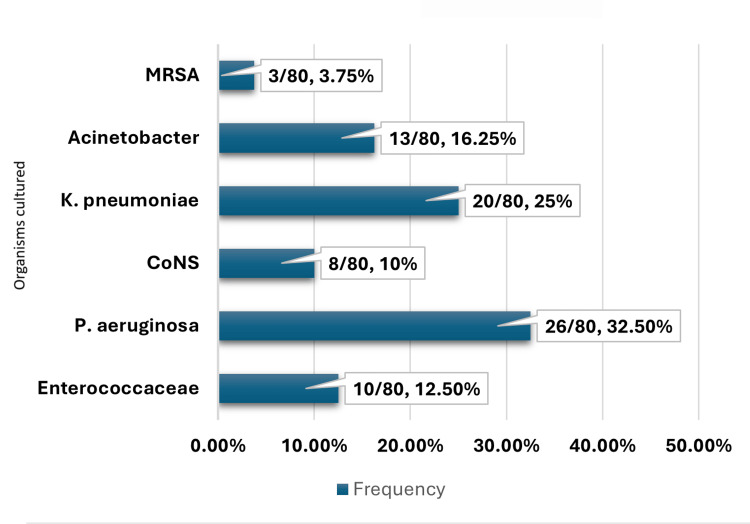
Distribution of various isolates cultured. Cultures isolated in the present study are as follows: (1) Enterococcaceae, (2) *Pseudomonas aeruginosa*, (3) CoNS, (4) *Klebsiella pneumoniae*, (5) Acinetobacter species, (6) MRSA. CoNS: coagulase-negative Staphylococci;MRSA: methicillin-resistant *Staphylococcus aureus*

*Pseudomonas aeruginosa* (26/80, 32.5%), *Klebsiella pneumoniae* (20/80, 25.0%), Acetobacteraceae (13/80, 16.25%), Enterococcaceae (10/80, 12.5%), coagulase-negativeStaphylococcaceae (8/80, 10.0%), and methicillin-resistant *Staphylococcus aureus*(3/80, 3.75%) were the organisms observed in the culture (Figure 1).

The antibiotic sensitivity of the cultured colonies to drugs such as penicillin, beta-lactams, macrolides, aminoglycosides, second-, third-, and fourth-generation cephalosporins, and fluoroquinolones, which are commonly used in the treatment of CRS, albeit with varying levels of effectiveness, was assessed in our study.

In general, variable sensitivity to third- and fourth-generation cephalosporins was observed in the study (based on minimum inhibitory concentration). Interestingly, the isolates exhibited 100% resistance to amoxicillin and clavulanic acid and macrolides, especially erythromycin and azithromycin. On the contrary, 100% sensitivity to aminoglycosides (gentamicin, tobramycin) and varying levels of sensitivity to ciprofloxacin and levofloxacin were also noticed. It was observed that 50/80 patients were sensitive to ciprofloxacin and 30/80 were sensitive to levofloxacin. The antibiotics were chosen based on the MIC, i.e., the drug with the least MIC (meaning the most effective drug) was chosen (Table 4).

The patients were started on appropriate antibiotics as per sensitivity for a course of two weeks. The patients improved symptomatically which was reflected in the significant reduction of Lund-Kennedy scores and overall SNOT-22 scores following antibiotic course across all age groups in both males and females (Tables 5, 6).

**Table 6:** Average SNOT-22 scores before and after antibiotics treatment. SNOT: Sino-Nasal Outcome Test

Age group (years)	Avg. SNOT-22 scores in males		p-Value	Avg. SNOT-22 scores in females		p-Value
	Before antibiotics	After antibiotics		Before antibiotics	After antibiotics	
18-25	31.00	29.72	0.008	29.77	26.44	0.002
25-45	28.40	26.63	0.001	31.22	29.22	0.001
45-65	28.09	26.36	0.008	34.00	32.00	0.004
>65	0	0	0	27.33	24.66	0.057

The patients were classified into the following three groups based on their SNOT-22 scores: mild, moderate, and severe which was akin to the classification proposed by Toma and Hopkins [15]. Most of the patients fell in the "moderate" category (76/80 95%) and few (4/80, 5%) had mild disease before initiation of treatment. Following antibiotic treatment, the number of patients with mild disease was 13/80 (16.25%) and the number of patients with moderate severity reduced to 67/80 (83.75%). In each group, there was a significant decrease (p≤0.005) in the average SNOT-22 score and Lund-Kennedy endoscopic scoring system across all age groups in both men and women.

## Discussion

Chronic rhinosinusitis is a disease of serious concern because of its worldwide occurrence. The signs and symptoms of the disease are facial pain, nasal discharge, and obstruction which cause significant morbidity to the patient. However, the etiopathogenesis of chronic rhinosinusitis is poorly understood. Many theories have been put forth to explain the etiology but with limited success. Nevertheless, the disease needs to be addressed and treated appropriately to provide relief to the patient. It has been sufficiently established in the literature that antibiotics have a greater role to play in the medical management of the disease and have proven effective like topical corticosteroids.

However, the differential efficacy of antibiotic treatment has been reported in the literature [12]. The present study clearly points out that the nasal microbiome in patients with chronic rhinosinusitis differs and consists of unique isolates with variable sensitivity to antibiotics, which may explain the poor response to treatment. Therefore, it is our conjecture that it is imperative to identify the nasal microbiome of the populace under treatment and choose appropriate antibiotic regimens that are suitable and tailored to the patient’s local microbiome. Empirical therapy may provide relief in patients with microbiomes that are sensitive to the chosen antibiotics but may fail to alleviate symptoms in microbiomes that are resistant to antibiotics.

Our study hypothesized that the nasal mucosa isolates cultured were the pathogenic invasive species disturbing the local microbiome. A broad-spectrum antibiotic may wipe out the entire nasal microbiota thereby paving the way for colonization of the sinonasal mucosa by new invasive species that may further cause acute exacerbations of the disease creating a vicious cycle. Therefore in an era of targeted therapies, antibiotic regimens directed towards the specific organism might yield better results in patients afflicted with chronic rhinosinusitis. The results obtained clearly indicated that there was a significant improvement in the disease severity before and after a specific antibiotic regimen. Therefore, tailor-made antibiotic regimens may offer better treatment outcomes in patients with chronic rhinosinusitis and may even mitigate the need for surgery in some cases.

Chronic rhinosinusitis is a disease with multifarious etiology but none of them fully explains the etiopathogenesis of the disease [16-21]. Although the nasal microbiome has not yet been fully explored and understood, the microbiomes of other systems have been studied in greater detail, especially those of the gastrointestinal tract and skin, and the same theory can be extrapolated to the nasal cavities. This study states that the system is colonized by a local microbiome that acts as commensals and secretes antimicrobial proteins and lipid by-products which suppress the growth of pathogenic flora. Any external change including surgeries or inappropriate antibiotic usage may alter the microbiome and induce chronic inflammation.

Ongoing research focuses on culture-independent molecular sequencing studies to identify clusters of flora that may as a whole function as drivers of inflammation. The pretext is that the nasal microbiome may harbor flora that are non-culturable by traditional methods, a phenomenon rightfully termed "the great plate count anomaly." Many trials and treatment guidelines for chronic rhinosinusitis have been published to date, viz., the International Consensus Statement on Allergy and Rhinology: Rhinosinusitis 2021 (ICAR 2021), European Position Paper on Rhinosinusitis and Nasal Polyps 2020 (EPOS 2020), Chinese Society of Allergy and Chinese Society of Otorhinolaryngology-Head and Neck Surgery Guideline (CSA/CSO) 2020, Clinical Practice Guideline (Update): Adult Sinusitis (CPGAS) 2015, and Joint Task Force on Practice Parameters (JFTFPP) 2014, of which only the Joint Task Force on Practice Parameters (JFTFPP) recommends the use of antibiotics, especially macrolides, with oral corticosteroids [22-24]. None of the studies offer clear indications regarding the use of antibiotics, suggesting a dearth of resources that corroborate the use of a culture-based antibiotic regimen.

According to published literature, macrolides are as effective as corticosteroids in controlling chronic rhinosinusitis. The corollary being macrolides, apart from their action of inhibiting protein synthesis by inhibition of messenger RNA from translating new proteins, possess anti-inflammatory and immunomodulatory properties along with inhibition of mucus hypersecretion and activation of mucociliary function of the respiratory epithelium thereby may provide symptomatic relief in chronic rhinosinusitis [22]. However, as our study population demonstrates a 100% resistance to macrolides, administering macrolides would have borne little to no benefit. Therefore, it is our recommendation that tailoring the regimen to the patient can thus provide better treatment outcomes for chronic rhinosinusitis. The nasal microbiome varies greatly based on genetic variation and local microcosmic environment [4]. Thus, understanding our patients’ local microbiome may enable healthcare providers to tailor regimens suited to patients.

Limitations

The use of molecular markers for proteins and genomic methods for isolating bacterial species could potentially identify the full spectrum of bacterial pathogens. The patients were evaluated and included in the study as and when they presented to the outpatient department of our hospital. Therefore establishing a baseline for these patients is impossible. A much larger study population could have yielded better and more consistent results. A much longer follow-up could also help in recording recurrences, future responses to antibiotics, development of antibiotic resistance, and any further changes in the patients' microbiome.

## Conclusions

This study found that the local nasal microbiome of the participants consisted of Enterococcus, Acinetobacter, Pseudomonas, coagulase-negative Staphylococcus, and Klebsiella species with *Pseudomonas aeruginosa *as the predominant organism which showed maximum sensitivity to aminoglycosides. This study also suggests a reevaluation of our understanding of the nasal microbiome and antibiotic guidelines, which will further enable the development of case-specific antibiograms for chronic rhinosinusitis.
